# Impact of patient and treatment characteristics on heart and lung dose in adjuvant radiotherapy for left-sided breast cancer

**DOI:** 10.1186/s13014-019-1364-3

**Published:** 2019-08-28

**Authors:** Tobias Finazzi, Van-Trinh Nguyen, Frank Zimmermann, Alexandros Papachristofilou

**Affiliations:** Clinic of Radiotherapy and Radiation Oncology, University Hospital Basel, Petersgraben 4, 4031 Basel, Switzerland

**Keywords:** Breast cancer, Adjuvant radiotherapy, Organs at risk, Heart dose, Lung dose, Lymph nodes, Intensity-modulated radiotherapy

## Abstract

**Purpose:**

The heart and lungs are routinely exposed to incidental irradiation during adjuvant radiotherapy (RT) of breast cancer. We analyzed the impact of patient and treatment characteristics on heart and lung dose in left-sided breast RT.

**Methods:**

We analyzed 332 female patients treated with left-sided breast RT between 2013 and 2018. Mean heart dose (MHD), left mean lung dose (MLD) and heart / lung V_20Gy_ were collected from treatment plans. Patients were stratified by RT technique (3D-conformal RT, 3DCRT; intensity-modulated RT, IMRT; volumetric modulated arc therapy, VMAT) and target volumes, including lymph node RT (LN-RT). Patient characteristics (body mass index (BMI), heart and lung volume) were assessed using correlation analyses.

**Results:**

LN-RT was performed in 111 patients with increased MHD (median 4.6 vs. 3.3 Gy; *p* < .01), left MLD (14.8 vs. 7.7 Gy; *p* < .01) and left lung V_20Gy_ (30.0% vs. 14.4%; *p* < .01) compared to treatment without LN-RT. Internal mammary LN-RT further increased organ doses compared to RT involving only supraclavicular +/− axillary LN (*p* < .01 for all values; MHD 6.9 vs. 4.2 Gy). In 221 patients treated without LN-RT, IMRT/VMAT was associated with higher left lung doses (MLD 9.1 vs. 7.4 Gy, *p* < .01; V_20Gy_ 18.8% vs. 14.0%, *p* < .01) compared to 3DCRT. A negative correlation between total lung volume and both MHD (*r* = − 0.38; *p* < .01) and heart V_20Gy_ (*r* = − 0.37; *p* < .01), as well as a weak positive correlation of BMI and MHD (*r* = 0.27; *p* < .01) were observed.

**Conclusions:**

In adjuvant RT for left-sided breast cancer, LN-RT is associated with a marked increase in heart and lung doses, particularly with internal mammary LN-RT. Potential advantages of IMRT/VMAT for breast or chest wall RT need to be weighed against a moderately increased lung dose.

## Background

Adjuvant radiotherapy (RT) is a standard procedure after surgery for breast cancer, reducing the risk of locoregional recurrence and breast cancer death after breast conserving surgery, as well as after mastectomy in high-risk patients [1, 2]. Since most breast cancer patients are cured of their disease, potential long term hazards of RT need to be considered. In particular, incidental irradiation of the heart in left-sided breast RT has been linked with an increased risk of cardiac events [3–5]. In addition, radiation dose to the lung harbors the risk of radiation pneumonitis, lung fibrosis and secondary lung cancer [4, 6, 7].

Although improved RT techniques can potentially better spare organs at risk (OAR), heart and lung dose remain important dosimetric surrogates for long term effects and hence influence clinical decision making in adjuvant RT for (left-sided) breast cancer. We analyzed the impact of patient and treatment characteristics on heart and lung dose in a contemporary cohort of patients treated with left-sided breast RT, aiming to better quantify potential relationships and allow for a more refined consideration of these factors in clinical practice.

## Materials and methods

We retrospectively identified female patients treated with adjuvant RT for left-sided breast cancer (including ductal and lobular carcinoma in situ) in our institution between 1st April 2013 and 31st August 2018. Exclusion criteria were previous irradiation of the left breast, indications other than adjuvant (e.g. palliative) RT, partial breast irradiation, uncommon fractionation (single doses other than 1.8–2.67 Gy), and documented refusal of data collection for scientific purposes. This analysis was approved by the Ethics Committee Northwest and Central Switzerland (EKNZ).

Data on patient and treatment characteristics were collected from electronic medical records in MOSAIQ® (Elekta, Stockholm, Sweden) and ISMed© (ProtecData AG, Boswil, Switzerland). For each patient, size and weight were noted for calculation of the body mass index (BMI; kg/m^2^). Individual heart and lung volumes (cm^3^) were documented from planning computed tomography (CT) data in stored RT plans. Treatment details were collected for each patient; this included RT technique, target volume and dose fractionation. Patients were generally treated in supine position with both arms above the head. Noted RT techniques were 3-dimensional conformal radiation therapy (3DCRT), intensity-modulated radiation therapy (IMRT), and volumetric modulated arc therapy (VMAT). When regional lymph nodes (LN) were irradiated, regions were noted separately as axillary, supraclavicular and internal mammary nodes (IMN), with axillary and supraclavicular LN typically representing levels 1–3 and level 4, respectively, according to ESTRO consensus guideline, and the IMN extending caudally to the 4th – 5th rib [8].

For each patient, radiation dose to the heart and lung was collected from RT plans. Noted parameters, based on literature [3, 4, 6, 9] and feasibility of collection for all patients, were mean heart dose (MHD; Gy), left mean lung dose (MLD; Gy), as well as V_20Gy_ (%) of the heart and left lung. In case of multiple RT plans, e.g. when LN regions were treated to a lower dose than the breast or chest wall, doses were collected from sum plans or summed up manually. In case of hypofractionated RT, due to the lower nominal prescription dose, MHD and left MLD were adjusted to a total dose of 50 Gy for the purpose of comparative analysis, and the V_16Gy_ was documented as a physical dose equivalent (40% of prescribed dose) to V_20Gy_ in conventional fractionation. Boost plans to the tumor bed were ignored for this study.

Statistical analysis was performed using RStudio v1.1.456 (Boston, USA). Group comparisons were performed to analyze heart and lung doses for different RT techniques and target volumes, using two-tailed t-test and Wilcoxon rank sum test. The relationship between patient characteristics (BMI, heart and total lung volume) and radiation exposure of the heart and lung was assessed using Spearman’s rank correlation coefficient. A *p*-value < .05 was considered to be statistically significant.

## Results

### Treatment characteristics

A total of 332 female patients treated with left-sided breast RT were eligible for analysis. Median age at time of RT was 59 years (range, [28–91]). Patients received a median dose of 50 Gy to the breast after breast-conserving surgery (*n* = 272), or to the chest wall after mastectomy (*n* = 60). Fractionation schemes were 50 Gy in 25 fractions (*n* = 119), 50.4 Gy in 28 fractions (*n* = 101), and 39.9 Gy in 15 fractions (*n* = 112). Irradiation of regional LN was performed in 33% of cases (*n* = 111), most commonly (80%) delivered with single doses of 1.8 Gy to a total dose of 45–50.4 Gy.

**Fig. 1:**
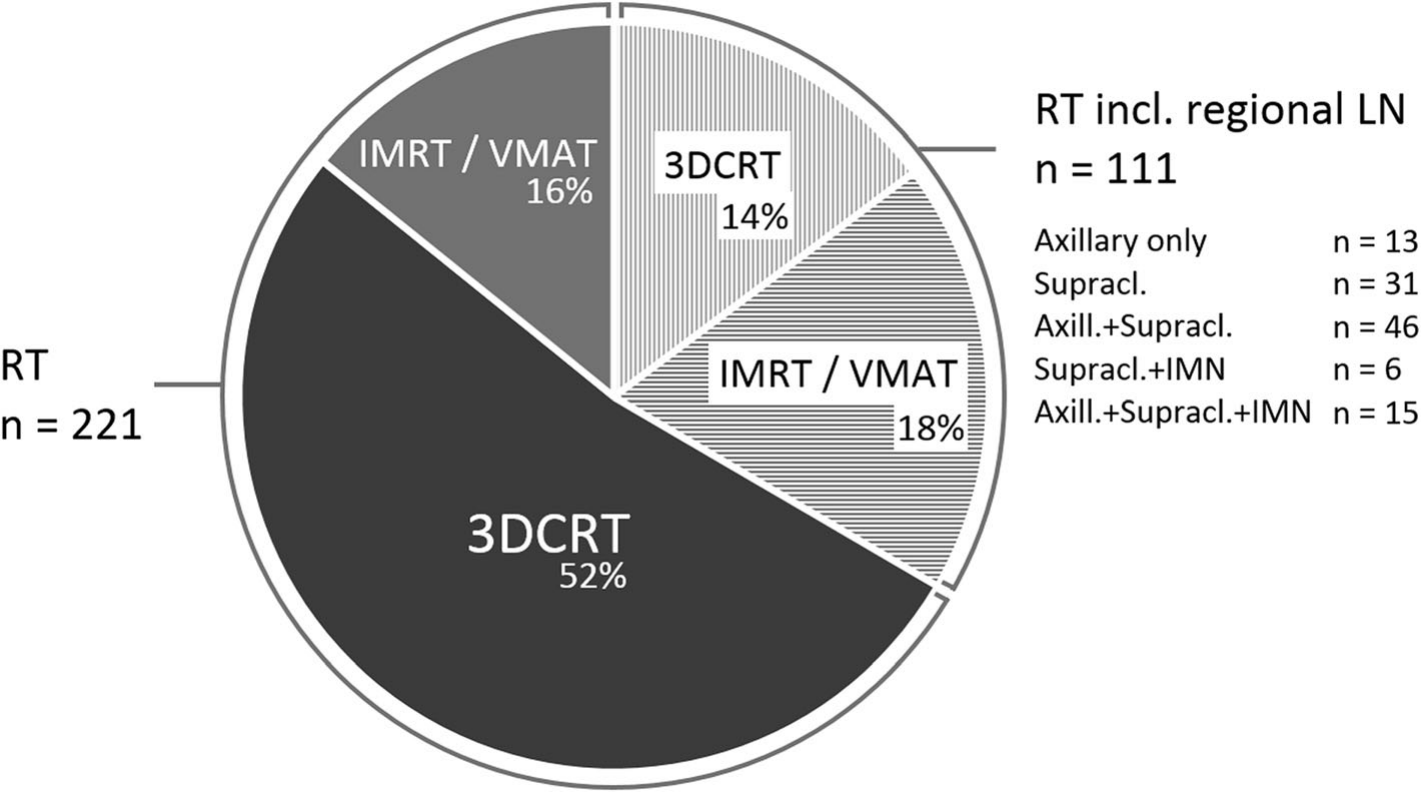
Summary of treatment characteristics for all patients treated with adjuvant RT for left-sided breast cancer (*n* = 332). *Abbreviations: RT, radiotherapy. 3DCRT, 3-dimensional conformal radiation therapy. IMRT, intensity modulated radiation therapy. VMAT, volumetric modulated arc therapy. LN, lymph nodes. Axill., axillary. Supracl., supraclavicular. IMN, internal mammary nodes*

Main treatment characteristics are summarized in Fig. 1. Overall, two-third (67%) of patients were treated with 3DCRT (*n* = 223), whereas one third (33%) received IMRT (*n* = 29) or VMAT (*n* = 80). As indicated in Fig. 1, regional LN-RT more often involved usage of IMRT/VMAT compared to cases without LN-RT (IMRT/VMAT in 55.9% vs. 21.3% of cases, respectively). When regional LN were treated, target volumes included the axillary LN (66.7%), supraclavicular LN (88.3%), and IMN (18.9%), either exclusively or in combination.

Institutional time trends for delivery of left-sided breast radiotherapy are presented in Table 1. The rate of patients receiving LN-RT increased over time, with 25.0% of patients receiving LN-RT in 2013–2014, compared to 37.5% in 2015–2018. For the whole cohort, usage of IMRT/VMAT also increased during the observation period, with an overall IMRT/VMAT rate of 5.6% in 2013–2014, compared to 46.0% in 2015–2018.

**Table 1:** Institutional time trends for left-sided breast radiotherapy

		2013–2014	2015–2016	2017–2018
Patients		108	131	93
Rate of LN-RT		25.0%	35.1%	40.9%
Rate of IMRT/VMAT use	Total (*n* = 332)	5.6%	21.4%	80.7%
	Treatment with LN-RT (*n* = 111)	22.2%	43.5%	94.7%
	Treatment without LN-RT (*n* = 221)	0.0%	9.4%	70.9%

Institutional time trends observed for delivery of left-sided breast radiotherapy. A marked increase in both regional LN-RT, and the use of IMRT/VMAT, was observed during the period of our study. Although mostly used for treatments involving regional LN-RT, IMRT/VMAT also became a common delivery technique for left-sided breast or chest wall RT, due to presumed benefits for dose homogeneity and conformity

Abbreviations*: LN-RT* lymph node radiotherapy*; IMRT* intensity-modulated radiation therapy*; VMA,* volumetric modulated arc therapy

### Impact of patient characteristics on heart and lung dose

Patient characteristics, as well as heart and lung doses for the whole cohort, are summarized in Table 2. Median heart and total lung volumes were 495.6 cm^3^ (range, [271.6–1031.7]) and 2738.6 cm^3^ (range, [1591.3–5544.5]), respectively. Patient size and weight were available in 318 cases, resulting in a median BMI of 25.3 kg/m^2^ (range, [16.3–54.7]). For the whole cohort of 332 patients, MHD and heart V_20Gy_ were a median of 3.7 Gy and 3.1%, respectively. Left lung mean dose and V_20Gy_ were a median of 9.1 Gy and 18.1%.

**Table 2:** Patient characteristics and organ at risk doses

		Median	Min	5th percentile	95th percentile	Max
Age at time of radiotherapy		59	28	39	80	91
Patient anatomy						
	Heart volume	495.6 cm^3^	271.6 cm^3^	353.6 cm^3^	696.9 cm^3^	1031.7 cm^3^
	Lung volume	2738.6 cm^3^	1591.3 cm^3^	1991.5 cm^3^	4155.6 cm^3^	5544.5 cm^3^
	Body mass index	25.3 kg/m^2^	16.3 kg/m^2^	19.1 kg/m^2^	36.5 kg/m^2^	54.7 kg/m^2^
Organ at risk doses						
	Heart mean dose (Gy)	3.7 Gy	0.4 Gy	1.1 Gy	7.4 Gy	14.8 Gy
	Heart V_20Gy_ (%)	3.1%	0.0%	0.0%	10.3%	39.0%
	Left lung mean dose (Gy)	9.1 Gy	1.0 Gy	4.5 Gy	17.4 Gy	19.6 Gy
	Left lung V_20Gy_ (%)	18.1%	0.0%	7.4%	37.0%	49.6%

Summary of patient characteristics and organ at risk doses for all patients treated with adjuvant radiotherapy for left-sided breast cancer (*n* = 332)

**Fig. 2:**
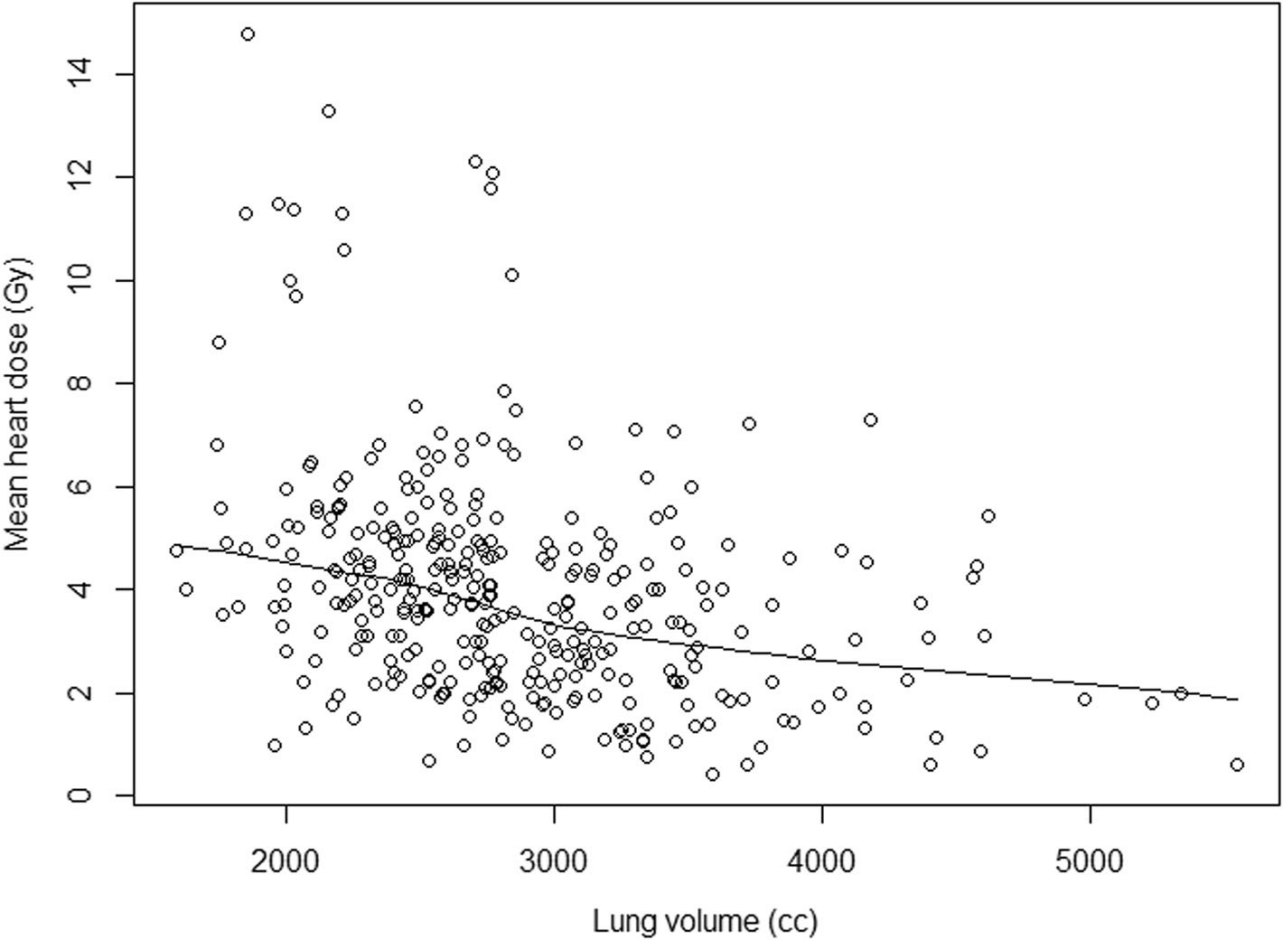
Scatter plot of total lung volume (cc) and mean heart dose (Gy) for all patients (*n* = 332). The fitted LOESS curve illustrates a weak to moderate negative correlation between lung volume and mean heart dose. *Abbreviations: cc, cubic centimeter. LOESS, locally estimated scatterplot smoothing*

Overall, no strong correlation between patient anatomy and heart or lung dose was observed. Dose exposure of the left lung (MLD, V_20Gy_) was not shown to be related to BMI, heart or lung volume. However, correlation analysis revealed a weak to moderate negative association between total lung volume and both MHD (*r* = − 0.38; *p* < .01; shown in Fig. 2) and heart V_20Gy_ (*r* = − 0.37; *p* < .01). This negative correlation was also seen in a separate analysis of patients with and without LN-RT, with similar effect size (*r* = − 0.36 to − 0.41; *p* < .01). In addition, a weak positive correlation of BMI and MHD (*r* = 0.27; *p* < .01) was observed. This was verified separately for cases without LN-RT (*r* = 0.34; *p* < .01), although no such correlation was seen when LN-RT was performed (*r* = 0.15; *p* = 0.12).

### Impact of treatment characteristics on heart and lung dose

**Fig. 3:**
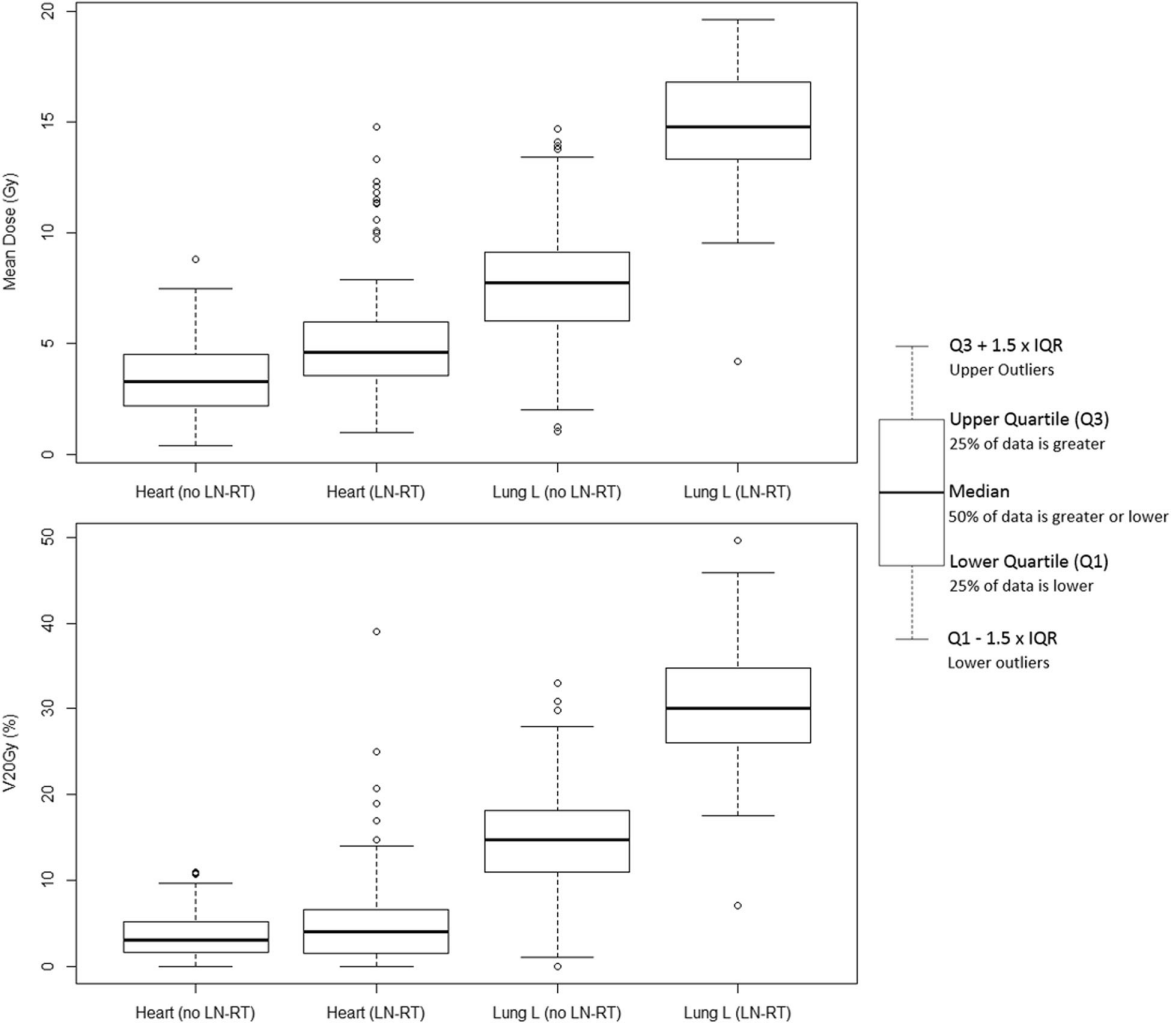
Box-and-whisker plots of heart and lung doses in patients treated with (*n* = 111) and without (*n* = 221) regional LN-RT. LN-RT increases mean doses to the heart and particularly the left lung (upper panel). LN-RT also increases V_20Gy_ for the left lung, with a corresponding trend for the heart (lower panel). Outliers, which are observations that fall at least 1.5 interquartile ranges outside of the box, are overall rare and mainly seen for the heart in some cases with LN-RT. *Abbreviations: LN-RT, lymph node radiotherapy. L, left. IQR, interquartile range*

Box-and-whisker plots in Fig. 3 illustrate the impact of regional LN irradiation on OAR dose. Regional LN-RT significantly increased MHD (median 4.6 vs. 3.3 Gy; *p* < .01), left MLD (14.8 vs. 7.7 Gy; *p* < .01) and V_20Gy_ of the left lung (30.0% vs. 14.4%; *p* < .01), with a trend for increased heart V_20Gy_ (4.0% vs. 3.0%; *p* = .06), compared to RT of the breast or chest wall without LN-RT. In particular, RT involving the IMN further increased heart dose compared to RT involving only the supraclavicular +/− axillary LN (median MHD 6.9 vs. 4.2 Gy, *p* < .01; heart V_20Gy_ 8.0% vs. 3.0%, *p* < .01). Radiation dose to the lung was also higher when IMN-RT was performed, compared to treatment of only the supraclavicular +/− axillary LN (left MLD 16.9 vs. 14.5 Gy, *p* < .01; left lung V_20Gy_ 33.0% vs. 30.0%, *p* < .01).

**Fig. 4:**
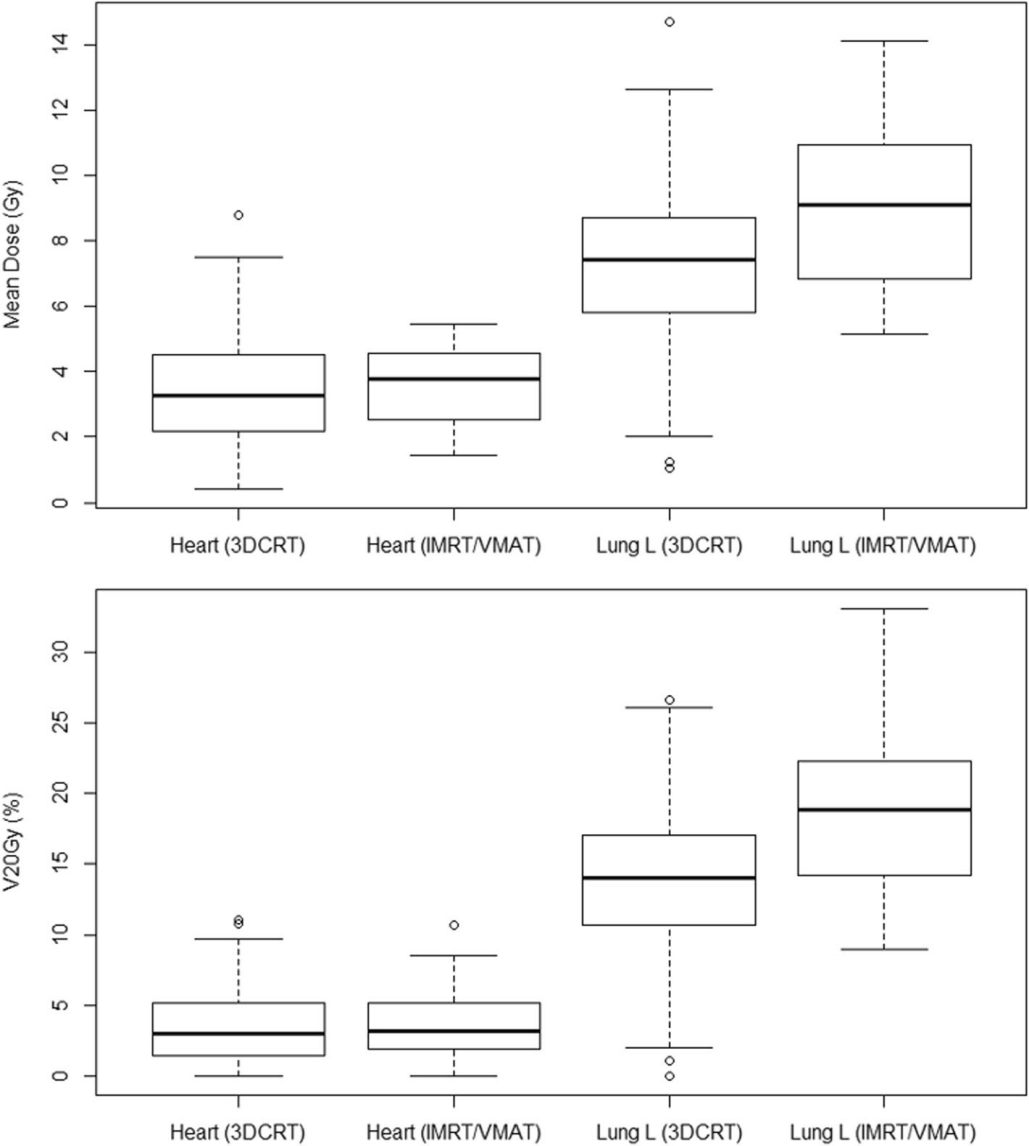
Box-and-whisker plots of heart and lung doses for patients treated to the breast or chest wall without regional LN-RT, using either 3DCRT (*n* = 174) or IMRT/VMAT (*n* = 47). A significant increase in lung doses can be seen for patients treated with IMRT/VMAT, whereas the difference in heart doses is non-significant. *Abbreviations: LN-RT, lymph node radiotherapy. 3DCRT, 3-dimensional conformal radiation therapy. IMRT, intensity modulated radiation therapy. VMAT, volumetric modulated arc therapy. L, left*

Overall, patients treated with IMRT or VMAT exhibited a higher MHD (median 4.5 vs. 3.3 Gy; *p* < .01), left MLD (13.6 vs. 8.1 Gy; *p* < .01) and V_20Gy_ of the left lung (26.4% vs 15.7%, *p* < .01) compared to 3DCRT. A subgroup analysis of patients treated without LN-RT (*n* = 221) was performed. As illustrated in Fig. 4, a moderate increase with IMRT/VMAT compared to 3DCRT remained for left MLD (median 9.1 vs 7.4 Gy; *p* < .01) and V_20Gy_ of the left lung (18.8% vs 14.0%; *p* < .01), whereas no difference in heart doses was observed.

## Discussion

We report on the impact of patient and treatment characteristics on heart and lung dose in a contemporary cohort of patients treated with left-sided breast RT. In summary, our results show that regional LN irradiation, and particularly RT of internal mammary LN, significantly increases heart and lung dose. Use of IMRT/VMAT moderately increases dose exposure of the left lung in RT of the breast or chest wall, whereas no strong correlation between patient anatomy and heart or lung dose was seen.

The observation of a higher heart and lung dose when treating the regional LN is not surprising. However, studies analyzing the magnitude of increase in contemporary clinical practice are limited. The clinical significance of accurately assessing this correlation stems from a more favorable view of regional LN-RT in many centers as well as clinical guidelines [10–12], following demonstration of improved outcomes in randomized trials [13–15].

The role of (left-sided) IMN-RT is particularly debated, mainly due to concerns about heart doses. In a randomized trial using 2-dimensional RT techniques, IMN-RT did not improve overall survival (OS), although a corresponding trend was observed [15, 16]. More recently, a prospective cohort study demonstrated increased OS for node-positive patients treated with IMN-RT [17]. In our cohort, patients treated with IMN-RT exhibited the highest heart and lung doses, with an average MHD of 8.3 Gy. This is almost identical to an average MHD of 8.4 Gy reported for left-sided IMN-RT in a systematic review [18]. Notably, our median MHD was lower than the mean, reflecting an observation of excessive doses in some cases with LN-RT (Fig. 3). In modern breast RT, the rate ratio (RR) of cardiac mortality has been estimated to increase by 0.04 per Gy MHD [4]. In our cohort, IMN-RT would therefore be associated with a RR of 1.33 of age-dependent cardiac mortality, compared to 1.18 when treating only the supraclavicular +/− axillary LN. These presumed risks of IMN-RT can be weighed against an absolute 8-year OS benefit of 3.7% in the aforementioned cohort study, which notably observed an equal number of cardiac deaths in patients receiving LN-RT with and without IMN-RT [17]. Contrary to some reports [19, 20], when excluding all types of LN-RT, we did not see an increase in MHD when IMRT or VMAT was used to treat the breast or chest wall. This may be a consequence of inverse treatment planning with priority given to heart sparing in left-sided breast radiotherapy, although lung doses should also be critically evaluted in treatment planning.

We observed a remarkable difference in lung doses, with regional LN-RT doubling both MLD and V_20Gy_ of the left lung. Previous analyses have shown a significant impact of regional LN-RT, as well as IMRT use, on lung doses [7]. Considering the known risk of secondary lung cancer, as well as cardiac mortality, smoking cessation should be considered a necessity for these patients [4]. For patients receiving RT only to the breast or chest wall, the use of IMRT/VMAT was associated with a moderate increase in lung doses in our cohort. This may be explained by patient selection, since IMRT/VMAT was often used in challenging cases for which tangential fields were deemed unsuitable, such as large breasts or patients with a sunken chest (pectus excavatum). Still, when considering use of intensity-modulated RT techniques, a presumed benefit in dose homogeneity, conformity and target coverage needs to be weighed against potentially increased low-dose exposure, as well as workload and costs, on an individual basis [21, 22]. Similarly, factors affecting cosmesis and quality of life, such as lymphedema, as well as the more short-term risk of radiation pneumonitis, may outweigh the risk of late effects in RT planning, depending on patient age and comorbidities [21, 23]. Future studies will therefore need to systematically address long-term outcomes to assess the true benefits of different delivery techniques used for breast RT. Besides systematic recording of cardiac and pulmonary events, this includes evaluating the role of unintended lymph node irradiation with 3DCRT compared to IMRT/VMAT, as well as cosmesis and lymphedema-related issues.

While we analyzed dosimetric parameters that are commonly used in clinical practice, it is important to note that their role as clinical predictors of toxicity is still a matter of ongoing debate. A case-control study of women who received breast RT between 1958 and 2001 found that the rate of major coronary events increased linearly with the MHD by 7.4% per gray, although heart doses were estimated retrospectively [3]. In contrast, more recent results indicate that the absolute cardiac risk after (left-sided) breast RT is likely much more modest using modern techniques [4, 5, 19, 24–26], and the rates of radiation pneumonitis and pulmonary fibrosis are still low when regional LN-RT is performed [13, 14].

To better estimate particularly the risk for cardiac late effects, standardized contouring of cardiac substructures has been proposed to improve consistency and precision of dose reporting [27, 28]. However, this has not found widespread clinical adoption, and dose to all cardiac segments should be minimized [29]. More effort has therefore been focused on reducing doses to OARs, and particularly the heart, using techniques such as RT in deep inspiration breath hold or prone positioning, which both can reduce the MHD [18, 20, 30–32]. Results of randomized trials may also lead to an increased use of partial breast irradiation in patients with early breast cancer [33–35].

## Conclusions

In conclusion, we observed a marked increase in heart and lung doses when treating regional LN, and particularly the IMN, in a contemporary cohort of patients receiving left-sided breast RT. Use of IMRT/VMAT was associated with a moderate increase in lung doses when treating the breast or chest wall. These results add to the body of data on heart and lung exposure as a function of patient and treatment characteristics in contemporary breast RT, which may aid clinical decision making and help tailor personalized RT for these patients.

## Data Availability

The datasets used and/or analysed during the current study are available from the corresponding author on reasonable request.
